# Ultrasound-Guided Funicular Block: Ropivacaine Injection into the Tissue around the Spermatic Cord to Improve Analgesia during Orchiectomy in Dogs

**DOI:** 10.3390/ani11051275

**Published:** 2021-04-28

**Authors:** Vincenzo Cicirelli, Pasquale Debidda, Nicola Maggio, Michele Caira, Giovanni M. Lacalandra, Giulio G. Aiudi

**Affiliations:** Department of Veterinary Medicine, University of Bari ‘Aldo Moro’, 70121 Bari, Italy; pdebidda1975@gmail.com (P.D.); dottormaggio@gmail.com (N.M.); michele.caira@uniba.it (M.C.); giovannimichele.lacalandra@uniba.it (G.M.L.); giulioguido.aiudi@uniba.it (G.G.A.)

**Keywords:** orchiectomy, ropivacaine, analgesia, dogs, local analgesia

## Abstract

**Simple Summary:**

Orchiectomy is a common surgical procedure performed on small animals and postoperative analgesia is very important because pain causes various negative effects that prevent patient recovery. Our study aimed to compare the intraoperative ultrasound-guided funicular block with ropivacaine and general anaesthesia using hypnotic and/or analgesic drugs and the postoperative hemodynamic stability and pain scores. This study demonstrated that the use of ultrasound-guided funicular, using ropivacaine, guarantees long-lasting and high-quality analgesic coverage and minimises the post-surgical pain inevitably associated with orchiectomy. This study stemmed from a general trend towards increasing attention on postoperative pain after orchiectomy in dog. Since veterinarians are becoming more focused on relieving surgical pain, anaesthetists are expected to use better protocols that can minimise pain. In fact, acute postsurgical pain is of great interest due to potential risk of becoming chronic if not treated properly. The routine use of the intraoperative ultrasound-guided funicular block with ropivacaine block is considered a useful alternative for pain management for orchiectomy in dogs, which can be performed under sedation or superficial general anaesthesia. In addition, the intraoperative ultrasound-guided funicular block with ropivacaine block ensures satisfactory analgesic coverage that lasts for at least 6 h postoperatively.

**Abstract:**

Orchiectomy is a common surgical procedure performed on small animals, and it requires postoperative pain management despite its relative simplicity. This study aimed to evaluate the hemodynamic stability, intraoperative administration of additional hypnotic and/or analgesic drugs, and postoperative pain scores following the combination of ultrasound-guided injection of ropivacaine hydrochloride into the spermatic cord and infiltration by the same anaesthetic of the incisional prescrotal line (ROP) or general anaesthesia. Dogs in the ROP group showed greater intraoperative hemodynamic stability and lower pain scores than the control group. The locoregional approach used in this study proved effective in minimising the responses to the surgical stimulus and ensured adequate analgesia intra- and postoperatively. This method, called ultrasound-guided funicular block, allows orchiectomy to be performed under deep sedation without general anaesthesia.

## 1. Introduction

Orchiectomy is a common surgery performed on small animals, and humane concerns dictate the provision of adequate analgesia. Persistent pain initiates a catabolic cascade that may have negative effects on homeostasis and cause discomfort [1,2,3,4]. Locoregional blocks can help control pain as part of a multimodal analgesic strategy and reduce inhalational anaesthetic requirements during surgery [5,6] and postoperative opioid requirements [7]. Locoregional anaesthesia can completely block the transmission of pain (in conscious patients) or nociceptive signals (in anaesthetised patients) and provide adequate analgesia [7]. The administration of local anaesthetics into the testicular parenchyma to provide analgesia during orchiectomy has been described. We aimed to compare the intraoperative ultrasound-guided funicular (UGF) block with ropivacaine and general anaesthesia using hypnotic and/or analgesic drugs and the postoperative hemodynamic stability and pain scores. Therefore, we hypothesised that the UGF block improves analgesia during canine orchiectomy without any side effects.

## 2. Materials and Methods

### 2.1. Animals

This study involved 50 young (<5 years old) mixed-breed dogs weighing 13–20 kg. They were of good health, had no previous pathologies, and were allocated to the very low aesthetic risk class (ASA 1). This study was performed at the Obstetric, Gynecological, and Andrological Clinic of the Veterinary Hospital of the Veterinary Medicine Department of the ‘Aldo Moro’ University of Bari. The dogs were selected for elective orchiectomy, and informed consent was obtained from their owners. The dogs were randomly assigned to two groups: the ROP and control groups.

### 2.2. Ethics

This study was performed following the ethical guidelines of the animal welfare committee. The Institutional Review Board approval of the study was obtained from the ‘Aldo Moro’ University of Bari. All animal procedures were performed following good veterinary practices for animal welfare according to the national laws in force (D. Lgs 116/92).

### 2.3. Pre-Surgery Procedure

In both groups, the dogs were sedated using intramuscular injections of 3 mcg/kg dexmedetomidine (Dexdomitor^®^, Vetoquinol Italia SRL, Bertinoro, Italy) and 0.25 mg/kg methadone (Semfortan^®^, Eurovet Animal Health BV, Bladel, The Netherlands) [8,9] mixed in the same syringe. When the dogs were sedated, a 20-G venous catheter was inserted to start a standard maintenance fluid therapy. From this point throughout the surgery, the following vital parameters were instrumentally monitored: heart rate, electrocardiographic trace, pulse oximetry, non-invasive blood pressure, and body temperature (monitor GE-Datex Ohmeda B 450) [10]. Trichotomy and surgical scrub were performed [11].

### 2.4. ROP Group Protocol

**Figure 1:**
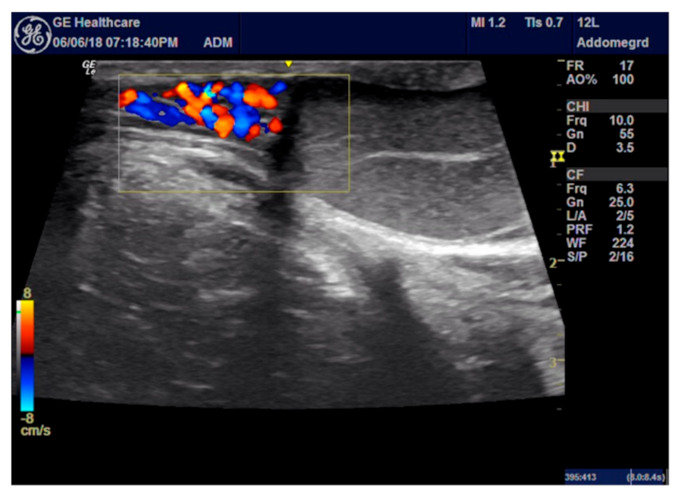
Ultrasound system images for procedural guidance.

**Figure 2:**
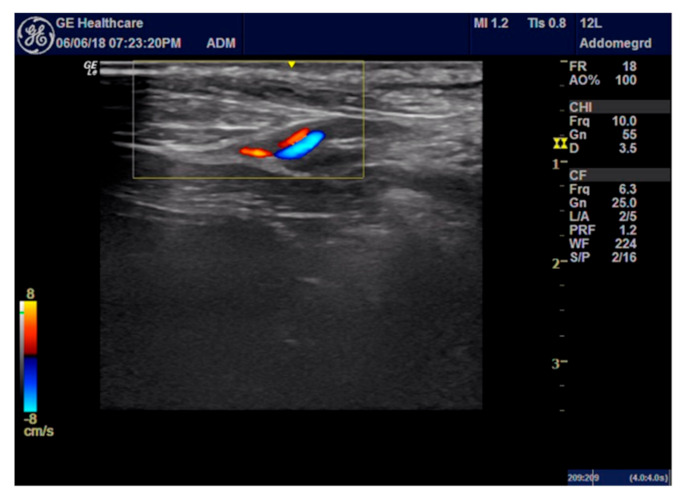
The anaesthetised spermatic cord at the superficial inguinal ring is shown.

**Figure 3:**
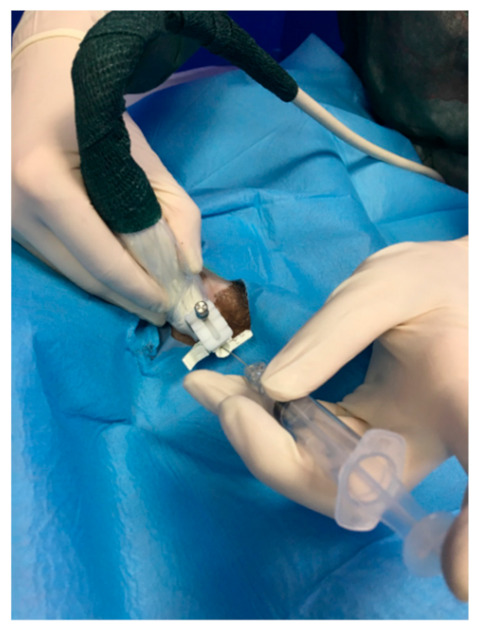
The ultrasound-guided peripheral nerve block by injection of a solution containing 0.5% concentrated ropivacaine hydrochloride into the spermatic cord at the level of its emergence from the superficial inguinal ring.

**Figure 4:**
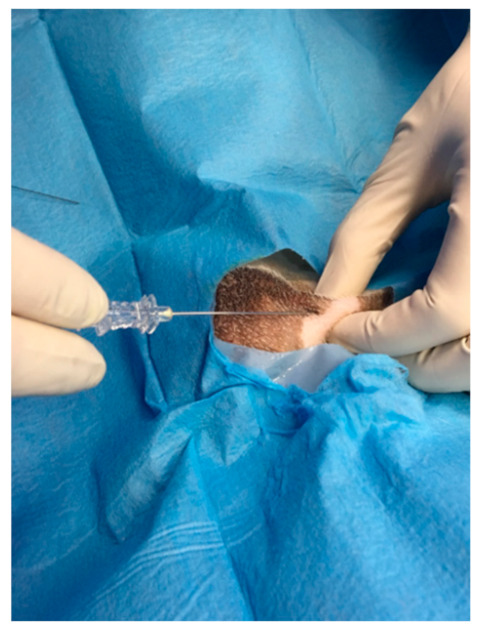
Infiltrative injection of ropivacaine hydrochloride concentrated at 0.5% at the prescrotal incisional line.

For the ROP group, a peripheral nerve block was performed under ultrasound-guided (GE Healthcare Logiq E) injection using a BD spinal anaesthesia needle with a Quincke tip (22 G, 0.7 × 90 mm) (Figure 1 and Figure 2). Ropivacaine hydrochloride (0.2 mL/kg at 0.5% concentrate) (Naropina^®^, Aspen Pharma Trading 69 Limited) was injected into the spermatic cord in two locations: at the level of its emergence from the superficial inguinal ring (Figure 3) and in the prescrotal incisional line (Figure 4).

### 2.5. Control Group Protocol 

In the control group, propofol (Vetofol^®^, Esteve, Barcelona, Spain) at 2 mg/kg was administered intravenously to induce general anaesthesia 20 min after sedation [12].

### 2.6. Surgery Procedure

The animals were placed in dorsal decubitus on an insulating mat to minimise thermal dispersion. All dogs were operated on by the same team, and all interventions lasted approximately 20 min, with a prescription approach for all animals [13,14,15]. Before the surgery, the heart rate and mean arterial pressure values were recorded, for both groups, as bases for the subsequent evaluation of the cardiovascular response to surgery (preincisional values). In the event of an increase in heart rate or blood pressure (>30% compared with preincisional values) during the procedure in response to surgical pain, a bolus of fentanyl was administered [16] intravenously at 2 mcg/kg (Fentadon^®^, Eurovet Animal Health BV). A bolus of propofol (1.0–2 mg/kg) was administered intravenously for the recurrence of motor responses and the wearing-off of sedation for some cases. Other boluses of fentanyl and/or propofol were administered for insufficient analgesia or motor responses, respectively. At the end of the procedure, 0.2 mg of Meloxicam^®^ (Metacam, Boehringer Ingelheim Italia S.p.A.) was administered subcutaneously [17,18] in all animals in this study. Adequate assistance was provided until the righting reflex was observed.

### 2.7. Postoperative Pain Evaluation

During the postoperative period, and immediately after the reappearance of the righting reflex and the assumption of sternal decubitus, postoperative pain and discomfort were assessed using the Glasgow CMPS-SF at 1-h intervals for 6 h. Animals who had a score of 4 on the pain scale were administered relief analgesic therapy (methadone IM at a dosage of 0.2 mg/kg) [11]. The postoperative pain score was determined by a different operator who was blinded to the group the dog belonged to.

### 2.8. Data Analysis

The data collected using forms were entered into a database created with an Excel spreadsheet, and data analysis was performed using Stata MP16 software. The skewness and kurtosis tests were used to evaluate the normality of the continuous variables; a normalisation model was set up to normalise those not normally distributed using the logarithmic function. Continuous variables were presented as mean (standard deviation [SD]) and range, and categorical variables were presented as proportions. The repeated-measures mixed model test was used to compare the continuous variables of the groups and the detection times, using age as a random factor; the χ2 and Fisher’s exact tests were used to compare the proportions. For all the tests, 2-sided *p*-values of <0.05 were considered indicative of statistical significance.

## 3. Results

### 3.1. ROP Group

None of the 25 dogs in the ROP group required emergency analgesics (fentanyl) intraoperatively. It was necessary to administer a bolus of propofol to 17 of them (variable dosage 1.0–1.5 mg/kg) to deepen the anaesthesia and facilitate the completion of the surgery. Moreover, two boluses of propofol were needed (dosage always variable between 1.0 and 1.5 mg/kg) for five dogs in the ROP group to safely complete the procedure. For three dogs, the level of sedation by premedication was adequate to complete the procedure without the administration of emergency or hypnotic drugs. During the postoperative period, none of the animals in the ROP group had a score exceeding 4 on the Glasgow pain scale; therefore, no postoperative rescue analgesia was administered. In this group, only three dogs had a score of 3 during the second hour of evaluation, while for all the others, the score fluctuated between 1 and 2 in 6 h of evaluation.

### 3.2. Control Group

All animals in the control group required a bolus of fentanyl because the surgical stimulation had affected important haemodynamic responses, with a >30% increase in heart rate, respiratory rate, and blood pressure compared with the basic parameters of anaesthesia.

For 16 animals, it was also necessary to administer a bolus of propofol (variable dosage of 1.0–2 mg/kg) to ensure adequate hypnosis to carry out the surgery. For the remaining nine animals in this group, two propofol boluses were administered to maintain analgesia (dosage of 1.5 mg/kg for the first bolus, and 1–1.5 mg/kg for the second bolus).

During the postoperative period, the score on the Glasgow scale exceeded the maximum limit (4 or more), at least once during the 6 h, for 24 animals in the control group, and an analgesic was administered. For one dog alone, the scores of the six assessments remained within the maximum limits. An analgesic was administered during the first hour of evaluation for two dogs, during the second hour for nine dogs, and during the third hour for 10 dogs. However, for three dogs, methadone was administered twice: during the second and sixth hours, the first and fifth hours, and the first and sixth hours, respectively.

A statistically significant difference in the fentanyl administrations in the control (100.0%; *n* = 25/25) and treatment (0.0%; *n* = 0/25; *p* < 0.0001) groups was observed; each animal belonging to the control group received a 2 mg/kg bolus. The observed events related to propofol administration are presented in Table 1.

ANOVA for repeated measures showed statistically significant differences between the Glasgow scale scores (*p* < 0.0001) and detection times (*p* < 0.00001) of the groups, and the interactions between time and the groups (*p* < 0.001; Table 1).

**Figure 5:**
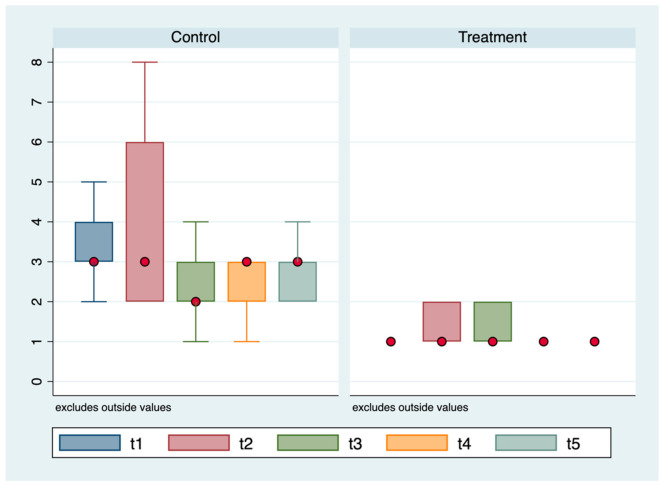
Glasgow CMPS-SF scale mean values according to the groups (treatment vs. control) and time of detection.

A statistically significant difference in methadone administration was observed in the control (96.0%; *n* = 24/25) and treatment (0.0%; *n* = 0/25; *p* < 0.0001) groups (Figure 5).

In the control group, 21/24 (87.5%) of the dogs received a bolus and 3/24 (12.5%) received two boluses. The first bolus of methadone was administered during the first hour for 4/24 (16.6%) cases, during the second hour for 10/24 (41.7%) cases, and during the third hour for 10/24 (41.7%) cases. The second bolus was administered during the fifth hour for 1/3 (33.3%) cases and during the sixth hour for 2/3 (66.7%) cases.

## 4. Discussion

This study did not cause major complications or death in any of the 50 dogs. The results of this study show that all animals were orchiectomised under anaesthesia with a good technique. The team of veterinarians who conducted this study ensured that all animals had the utmost attention for the provision of good analgesia. This study was motivated by the increasing attention being given to postoperative pain after spaying procedures in dogs [19]. This shows that veterinarians are becoming more careful in relieving surgical pain. For this reason, anaesthetists have adopted better protocols that can minimise pain, and, therefore, optimise surgical results. These techniques included the use of single drugs, and drug combinations with varying dosages, routes, and timing [19].

Multimodal analgesia was performed for the ROP group, which showed better analgesic and hypnotic results. The analgesic strategy used in this study, including the drugs administered both systemically and locally, was effective in providing pain relief [20]. None of the 25 dogs in the ROP group required emergency analgesics intra- and postoperatively. This study demonstrates that ropivacaine, injected into the inguinal ring close to the genito-femoral nerve, distributes rapidly into the spermatic cord, which improves perioperative analgesia for dogs undergoing orchiectomy. To exert its antinociceptive effect, a local anaesthetic must be proximally distributed to the point where noxious stimuli occur [7]. In addition, it was possible to perform orchiectomy in sedated animals that did not feel pain: this suggests that the UGF block is highly effective in blocking nocifensive autonomic responses to testicular surgery. However, sedation may not be sufficient in some cases: in both groups, it was necessary to administer one or two boluses of propofol to deepen the anaesthesia due to the recurrence of motor responses. Overall, the UGF block is relatively inexpensive and easy to administer. This block is useful in busy general practices because ropivacaine can be administered during the clipping and aseptic preparation of the surgical site. In addition, all the surgeries lasted for approximately 20 min. The analgesic block did not influence the duration of the surgery. In addition, no adverse effects, such as haematoma or haemorrhage formation, were noted in the ROP group. These results suggest that the UGF block is clinically useful for intra- and postoperative analgesia. The use of long-acting anaesthetics, such as ropivacaine, ensures satisfactory analgesic coverage that lasts for at least 6 h [21] postoperatively, and this guarantees superior quality recovery and management, which minimised postoperative pain and reduced the need to administer analgesics. For a complete assessment of pain, it was necessary to observe the animals for a few hours postoperatively. Furthermore, the UGF block used in the ROP group resulted in extremely low Glasgow pain scores 6 h postoperatively, and no analgesics were administered. Moreover, the use of the Glasgow scale allowed the determination of the specific moments the animals in the control group benefited from relief analgesic coverage while preventing unnecessary ‘blind’ administrations.

## 5. Conclusions

Routine use of the UGF block is considered desirable for orchiectomy in dogs, which can be performed under sedation or superficial general anaesthesia. In addition, the UGF block ensures satisfactory analgesic coverage that lasts for at least 6 h [21] postoperatively. These findings may provide the foundations for further investigations into the cases of dogs that cannot undergo general anaesthesia.

## Figures and Tables

**Table 1:** Group-wise management of the sample with propofol (treatment vs. control).

Variable	Control Group (*n* = 25)	ROP group (*n* = 25)	Total (*n* = 50)	*p*-Value
Propofol administration; *n* (%)	25 (100.0)	22 (88.0)	47 (94.0)	0.235
N. doses; *n* (%)				
1 2	9/25 (36.0)	4/22 (18.2)	13 (27.7)	0.173
Dosage dose 1 (mg/kg); *n* (%)				
1.0 1.5 2.0	1/25 (4.0) 22/25 (88.0) 2/25 (8.0)	6/22 (27.3) 16/22 (72.7) 0/22 (0.0)	7/47 (14.9) 38/47 (80.9) 2/47 (4.2)	0.025
Dosage dose 2 (mg/kg); *n* (%)				
1.0 1.5	7/9 (77.8) 2/9 (22.2)	2/4 (50.0) 2/4 (50.0)	9/13 (69.2) 4/13 (30.8)	0.317

## Data Availability

The data presented in this study are available on request from the corresponding author.
